# Velocity

**Published:** 1871-04

**Authors:** 


### VELOCITY.

| | H. | Min. | Sec. |
|---|---|---|---|
| 1 mile locomotive | | 1. | 00 |
| 1 mile running, Henry Perritt | | 1. | 42½ |
| 1 mile pacing, Pocahontas | | 2. | 17¾ |
| 1 mile trotting, Bonner's Dexter | | 2. | 18¾ |
| 2 miles trotting, Flora Temple | | 4. | 50½ |
| 4 miles running, Lexington | | 7. | 19¾ |
| 3 miles trotting, Dutchman | | 7. | 32¼ |
| 16 miles trotting, Prince | | 50. | 00¾ |
| 20 miles trotting, Trustee | | 59. | 35½ |
| 100 miles trotting, Conqueror | 8. | 55. | 00 |
| 100 miles, couple, Busk and Robin | 10. | 17. | 22 |

| | Miles. |
|---|---|
| Ocean steamers average per hour | 11 |
| River boats | 20 |
| Race-horse at the rate of | 30 |
| Bird | 60 |
| Hurricane | 80 |
| Sound | 804 |
| Earth round the sun | 68,000 |
| Light | 690,000,000 |
| Electricity | 1,000,000,000 |

A telegram could go to the sun in five minutes. If a cannon ball were shot from the earth towards the sun, and on the instant of the flash a telegram was sent to that effect, the inhabitants of the sun would have two months to get ready for it, but it would be ten years before they would hear the explosion. All have seen that light travels faster than sound.

But it is not wholesome to be in a hurry. Locomotives have been reported to have moved a mile in a minute for short distances. But locomotives have often come to grief by such great rapidity. Multitudes in their haste to get rich are ruined every year. The men who do things maturely, slowly, deliberately, are the men who oftenest succeed in life. People who are habitually in a hurry generally have to do things twice over. The tortoise beat the hare at last. Slow men seldom knock their brains out against a post. Foot-races are injurious to health, as are all forms of competitive exercises ; steady labor in the field is the best gymnasium in the world. Either labor or exercise carried to exhaustion, or prostration, or even to great tiredness, expressed by "fagged out," always does more harm than the previous exercise has done good. All running up-stairs, running to catch up with a vehicle or ferry-boat, are extremely injurious to every age, and sex, and condition of life. It ought to be the most pressing necessity which should induce a person over fifty to run twenty yards. Those live longest who are deliberate, whose actions are measured, who never embark in any enterprise without "sleeping over it," and who perform all the every-day acts of life with calmness. Quakers are a proverbially calm, quiet people, and Quakers are a thrifty folk, the world over.

